# Chromosome 1 Sequence Analysis of C57BL/6J-Chr1^KM^ Mouse Strain

**DOI:** 10.1155/2017/1712530

**Published:** 2017-04-09

**Authors:** Fuyi Xu, Tianzhu Chao, Yiyin Zhang, Shixian Hu, Yuxun Zhou, Hongyan Xu, Junhua Xiao, Kai Li

**Affiliations:** ^1^College of Chemistry, Chemical Engineering, and Biotechnology, Donghua University, Shanghai, China; ^2^Department of Biostatistics and Epidemiology, Medical College of Georgia, Augusta University, Augusta, GA, USA

## Abstract

The Chinese Kunming (KM) mouse is a widely used outbred mouse stock in China. However, its genetic structure remains unclear. In this study, we sequenced the genome of the C57BL/6J-Chr1^KM^ (B6-Chr1^KM^) strain, the chromosome 1 (Chr 1) of which was derived from one KM mouse. With 36.6× average coverage of the entire genome, 0.48 million single nucleotide polymorphisms (SNPs) and 96,679 indels were detected on Chr 1 through comparison with reference strain C57BL/6J. Moreover, 46,590 of them were classified as novel mutations. Further functional annotation identified 155 genes harboring potentially functional variants, among which 27 genes have been associated with human diseases. We then performed sequence similarity and Bayesian concordance analysis using the SNPs identified on Chr 1 and their counterparts in three subspecies, *Mus musculus domesticus*, *M. m. musculus*, and *M. m. castaneus.* Both analyses suggested that the Chr 1 sequence of B6-Chr1^KM^ was predominantly derived from *M. m. domesticus* while 9.7% of the sequence was found to be from *M. m. musculus*. In conclusion, our analysis provided a detailed description of the genetic variations on Chr 1 of B6-Chr1^KM^ and a new perspective on the subspecies origin of KM mouse which can be used to guide further genetic studies with this mouse strain.

## 1. Introduction

The Chinese Kunming (KM) mouse colony, the largest outbred mouse stock maintained by commercial dealers nationwide in China, has been widely used in pharmaceutical and genetic studies [1]. Unlike other outbred mice, KM mouse has a complex evolutionary history. In 1944 during the World War II, Swiss mice were initially introduced into Kunming, Yunnan Province, China, from the Indian Haffkine Institute by Professor Feifan Tang via the Hump route with the help of the American Volunteer Group [2]. These mice were named KM mice after their initial location in China. Because most other mouse strains were lost and mouse facilities were damaged during the World War II, KM mouse became the only laboratory mouse available afterwards. They were gradually distributed throughout most of the country for medical studies. However, despite the importance of this outbred mouse, its underlying genetic structure remains unclear.

According to the Mouse Genome Informatics (http://www.informatics.jax.org/), over one thousand quantitative trait loci (QTLs) have been mapped on mouse chromosome 1 (hereafter referred to as Chr 1) including large amounts of QTLs related to metabolism disorder. However, very few candidate genes have been identified partly because of the large QTL intervals. In order to fine map the metabolism disorder QTLs on Chr 1 and identify the candidate genes, we established a population of Chr 1 substitution mouse strains, in which C57BL6/J (B6) was the host strain, and one KM mouse, five inbred strains, and twenty-four wild mice captured from various locations in China were selected as the Chr 1 donors [3]. In order to dissect the genetic structure and variations of this population and better severe further genetic studies, we have resequenced 18 strains of this population including C57BL/6J-Chr1^KM^ (B6-Chr1^KM^) with next-generation sequencing technology [4].

In this study, we analyzed the genome sequence data from B6-Chr1^KM^ strain and identified 0.48 million single nucleotide polymorphisms (SNPs) and 96,679 indels on Chr 1, of which 6.4% SNPs and 16.3% indels were considered to be novel. Functional annotation suggested that 474 variants had deleterious effect on gene functions. In addition, we explored the KM mouse genetic structure by performing sequence similarity and Bayesian concordance analysis (BCA) on Chr 1. Results suggested that KM mouse was predominately originated from *Mus musculus domesticus* and part of the sequence was from *M. m. musculus.*

## 2. Materials and Methods

### 2.1. Animals

B6 and KM mice were purchased from Shanghai SLAC Laboratory Animal Co., Ltd., China. One male KM mouse was mated with female B6 to produce hybrid F1, followed by 8 generations of backcrossing with B6 using marker-assisted selection, then brother × sister mating to create a B6-Chr1^KM^ Chr 1 substitution strain [3]. All mice were maintained under specific pathogen-free (SPF) conditions according to the People's Republic of China Laboratory Animal Regulations, and the study was conducted in accordance with the recommendations of and was approved by the Laboratory Animal Committee of Donghua University.

### 2.2. DNA Sequencing

B6-Chr1^KM^ genomic DNA was extracted from tail tissue of a male mouse using an AxyPrep™ Multisource Genomic DNA Miniprep Kit (Axygen, Hangzhou, China) according to the manufacturer's protocol.

Purified genomic DNA was sheared and size selected (300–500 bp). Paired-end sequencing (2 × 125 bp) was carried out with an Illumina HiSeq 2500 instrument (Illumina Inc., San Diego, CA, USA) on two lanes by WuXi AppTec (Shanghai, China) according to the manufacturer's protocol.

### 2.3. Read Alignment

Raw reads were filtered using NGS QC toolkit v2.3 [5] to remove reads containing more than 30% low-quality (Q20) bases. Filtered reads were aligned to the C57BL/6J reference genome (December 2011 release of the mouse reference genome (mm10) from Ensembl) using BWA (version 0.7.10-r789) with 12 threads [6]. The resulting SAM file was converted to a binary format and sorted with SAMtools v1.1 [7], followed by the marking of duplicate reads using picard-tools v1.119 (http://picard.sourceforge.net). To improve SNP and indel calling, indel realignment was conducted with Genome Analysis Toolkit (GATK v3.3) [8].

### 2.4. SNP/Indel Identification and Annotation

SNPs and indels were called using SAMtools mpileup and BCFtools call functions [7], with the '-uf' and '-cv' parameters, respectively. To identify a high-quality variant data set, variants were filtered using the BCFtools filter and VCFtools varFilter function [9]. The following parameters were used: for BCFtools filter, '-g 10 -G 3 -i 'QUAL>10 && MIN(MQ)>25 && MIN(DP)>6 && MAX(DP)<199 && (DP4[2]+DP4[3])> 2', and for VCFtools varFilter, '-2 0'.

Ensembl Variant Effect Predictor tool (VEP, v78) [10] was used to characterize the SNPs and indels, and the algorithm SIFT was used to predict whether a missense variant would have a deleterious effect on a protein-coding gene.

### 2.5. Sequence Similarity Analysis

SNP information for WSB/EiJ (WSB), PWK/PhJ (PWK), and CAST/EiJ (CAST) was downloaded from the Mouse Genome Project (MGP) database of the Sanger Institute. The Chr 1 consensus sequence for each strain was constructed using the SAMtools consensus parameters. The repeat-masked B6-Chr1^KM^ Chr 1 sequence was divided into 1955 100 kb segments. The similarities of each segment with the corresponding segments in the WSB, CAST, and PWK were evaluated. Sliding window similarity analysis was also performed using 500 kb windows and 100 kb sliding intervals.

### 2.6. Phylogenetic Analysis

Phylogenetic analysis was conducted with the previously reported BCA method [11], with the *Rattus norvegicus* Chr 1 sequence (version rn5) downloaded from Ensembl used as the out-group. Briefly, consensus sequences from the WSB, PWK, and CAST strains were mapped to the alignment and gaps filled with Ns. Collinear segments were partitioned into 830 loci using a minimum description length algorithm with a default maximum cost.

### 2.7. Phylogenetic Tree Evaluation

Nexus files corresponding to the WSB-derived or PWK-derived regions were converted to FASTA files, and then a neighbor-joining phylogenetic tree was constructed using MEGA6 program [12]. Subsequently, 1000 bootstrap replicates were performed to generate branch support values.

## 3. Results

### 3.1. B6-Chr1^KM^ Genome Background

**Figure 1:**
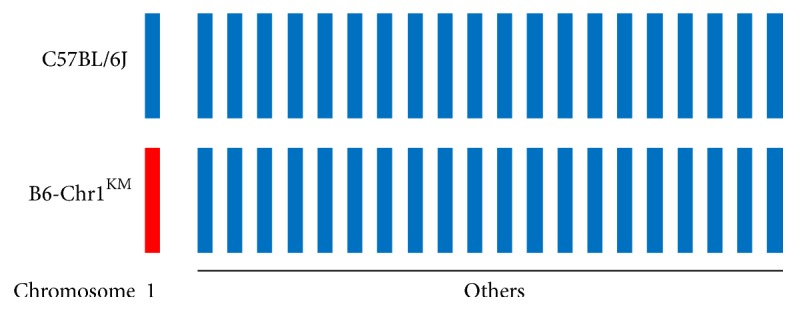
The characteristics of B6-Chr1^KM^ genome background. Blue bars represent B6 chromosome while the red represents KM mouse chromosome.

Chromosome substitution strains, also named as consomic strains, are designed to simplify the genome background and increase the power and speed of QTL mapping. The characteristic of consomic strain is that it only contains a single chromosome from the donor strain substituting the corresponding chromosome in the host strain. For B6-Chr1^KM^ consomic strain, Chr 1 sequence was derived from one KM mouse, while the genome background was from the B6 strain (Figure 1). In addition, sequences in the primary mouse reference assembly come from the same B6 strain. Therefore, our analysis of B6-Chr1^KM^ whole genome resequencing data only focused on Chr 1.

### 3.2. SNP and Indel Discovery

In this study, approximately one billion reads from the B6-Chr1^KM^ mouse strain were generated on two lanes of Illumina HiSeq 2500. A total of 78.65% of the reads were considered to be clean reads after quality control evaluation. Of them, more than 99% were aligned to the B6 mouse reference genome (mm10) using BWA with a mean genome-wide coverage of 36.6×.

A total of 479,956 SNPs and 96,679 indels were detected using SAMtools/BCFtools on Chr 1, in which 462,755 (96.42%) of the sites were homozygous. These variants were compared with variant calls from 36 key mouse strains from the Sanger Institute [13] as well as NCBI dbSNP142 variant data sets. This led to the identification of 449,089 SNPs (93.6%) as known, and the remaining 30,867 SNPs (6.4%) were classified as novel. For indels, 15,723 (16.3%) were classified as novel. In addition, we evaluated the variant calls using Sanger sequencing in our previous study which achieved high accuracy with 0.57% false positive and 0% false negative rate [4].

**Figure 2:**
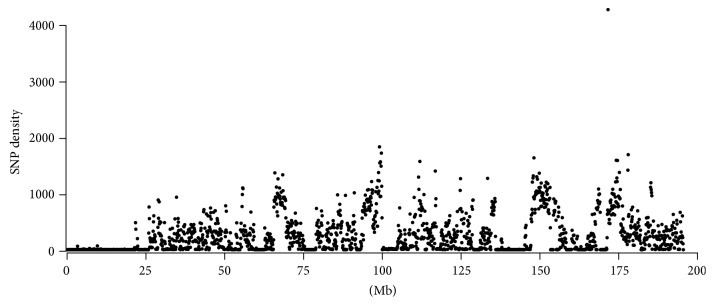
Distribution of SNP density on B6-Chr1^KM^ Chr 1. The SNP density is represented by the number of SNPs mapped within 100 kb physical intervals across Chr 1.

Next, we detected the distribution and density of SNPs over 100 kb window sizes. The observed average SNP density across the entire Chr 1 was 250 per 100 kb. However, different regions showed varying densities. For example, 29.5% of the Chr 1 sequence had an extremely low (0–5 SNPs per 100 kb) SNP density, while 9.1% had a high density (800 or more SNPs per 100 kb). The proximal region of Chr 1 was the longest region with a low SNP density encompassing nearly 25 Mb (Figure 2).

### 3.3. Functional Consequences of the SNPs and Indels

The putative consequences of SNPs and indels were cataloged using VEP from Ensembl (Table 1). The majority of the SNPs were located in intergenic (224,557, 18.7%) and intronic regions (575,013, 47.8%), and nearly 12% were classified as noncoding transcript variants. With regard to splice sites, 40 splice variants (including splice donor and splice acceptor variants) were found. The numbers of SNPs causing a premature stop codon or stop loss were 19 and 5, respectively. In addition, 2,378 (0.2%) missense variants were detected in 358 genes (one or more variants per gene). Among them, 380 variants (31.6%) from 113 genes were considered to have deleterious effects (SIFT < 0.05). Similar to the SNPs, the majority of indels were intronic (49.3%) and intergenic (17.1%) or within 5 kb upstream or downstream of a gene (16.9%). Only a small number of indels caused frameshift (22) and stop gain or loss (2). Among the novel variants, 7 caused a disruption of the translational reading frame; 10 were predicted as premature truncation of the protein due to gain or loss of stop codons; and 9 were located in splice donor regions. In addition, 104 novel missense variants from 20 genes had deleterious effects.

Next, we annotated these genes containing amino acid altering variants (SIFT < 0.05) and those with stop gain or loss, frameshift, and splice region variant genes with the Human-Mouse: Disease Connection database from Mouse Genome Informatics [14]. This analysis, which contained 155 genes, resulted in 27 genes associated with 49 different human disease-related phenotypes (Table 2), including macular degeneration, breast cancer, and immunodeficiency. Among these 27 disease genes, 9 have been investigated with mouse models, which had an in-depth phenotype information in different mouse genome background.

### 3.4. Sequence Similarity Analysis

**Figure 3:**
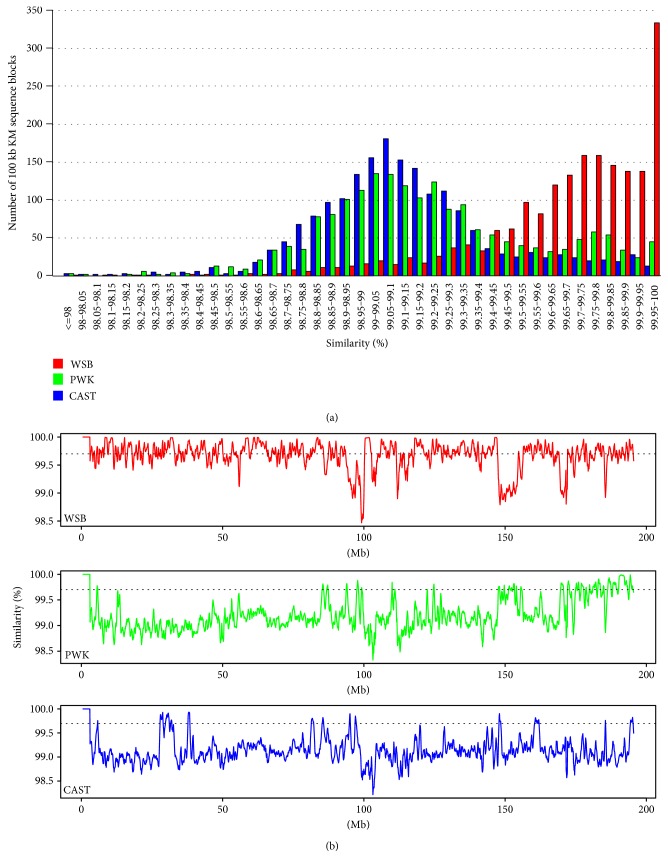
Sequence similarity between B6-Chr1^KM^ and WSB, PWK, and CAST Chr 1. (a) Distribution of the numbers of 100 kb blocks of the B6-Chr1^KM^ Chr 1 with sequence similarities (%) to the corresponding blocks of the WSB, PWK, and CAST Chr 1. (b) Sliding window analysis of the similarities of Chr 1 sequences between B6-Chr1^KM^ and WSB, CAST, or PWK. The B6-Chr1^KM^ Chr 1 sequence was compared using 500 kb windows and 100 kb sliding intervals. The horizontal line indicates the level of 99.7% sequence similarity.

The house mouse, *Mus musculus*, consists of three principal subspecies, with *M. m. domesticus* in Western Europe and the Middle East, *M. m. musculus* in Eastern Europe and Asia, and *M. m. castaneus* in Southeast Asia and India. Three genome sequences of the wild-derived inbred mouse strains, WSB, PWK, and CAST, which are broadly used to represent each of the subspecies, were selected for phylogenetic analysis. A Chr 1 consensus sequence was constructed for each strain using the SNP information from MGP. Because the simplest way to analyze phylogenetic divergence is by assessing sequence similarity, the Chr 1 sequence was separated into 1955 100 kb blocks and the similarities between each fragment and the corresponding sequences from WSB, PWK, and CAST were determined. The Chr 1 sequence was found to contain a large number of fragments with high sequence similarity to the corresponding sequence in WSB (Figure 3(a)), which is consistent with previous reports showing that KM mouse is derived from Swiss mice originated from the *M. m. domesticus* subspecies [1]. In addition, a bimodal distribution of blocks with two peaks of similarity was observed in a comparison of B6-Chr1^KM^ Chr 1 with PWK counterpart (Figure 3(a)). The first peak had only 99.05–99.1% sequence similarity to PWK, indicating the intersubspecies genome divergence of the Chr 1 sequence from *M. m. musculus*. The second peak had >99.7% sequence similarity to PWK (Figure 3(a)), indicating that the sequence of *M. m. musculus* introgressed into the KM mouse Chr 1. For the comparison of B6-Chr1^KM^ and CAST, we just observed one peak which suggested no signs of introgression of *M. m. castaneus* into the KM mouse Chr 1.

We next performed sliding window similarity analysis using 500 kb windows and 100 kb sliding intervals (Figure 3(b)). We found that 13.5% and 6.4% of the Chr 1 sequences had high similarity (>99.7%) with the corresponding sequences of PWK and CAST, respectively. The distal portion of the B6-Chr1^KM^ Chr 1 was found to have several regions that were highly similar to the corresponding regions of PWK with sharp boundaries between the regions of high and low similarity. However, we did not find any distinct boundaries between B6-Chr1^KM^ and CAST Chr 1 sequence.

### 3.5. Bayesian Concordance Analysis

**Figure 4:**
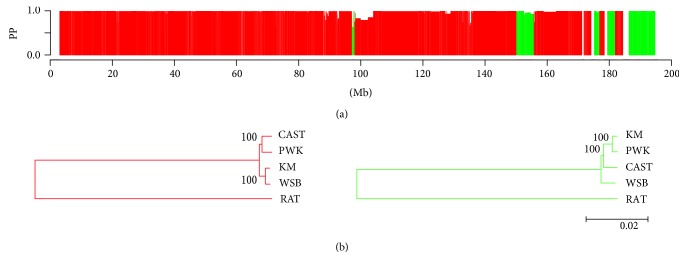
Phylogenetic analysis of B6-Chr1^KM^ Chr 1. (a) Fine-scale phylogenetic discordance of B6-Chr1^KM^ Chr 1 (PP indicates posterior probability). Red represents WSB, green indicates PWK, and white represents unknown. (b) Phylogenetic tree of the WSB-derived or PWK-derived sequences of B6-Chr1^KM^ and the wild-derived inbred mouse strain sequences. A neighbor-joining tree was generated using MEGA6 software. Red and green indicate regions supporting a single topology for KM/WSB and KM/PWK, respectively, which are both associated with a high posterior probability, as determined by BCA.

To determine the extent of phylogenetic discordance in B6-Chr1^KM^ Chr 1, we assessed the discordance along Chr 1 by BCA. A total of 886 partitioned individual locus trees were used to estimate Bayesian concordance factors. In BCA, 87.7% of the loci supported a single KM/WSB topology with higher posterior probability, and 9.7% supported a single KM/PWK topology. None of the loci supported a KM/CAST topology, and the remaining 2.6% had a complicated topology (Figure 4(a)). Highly conserved genomic regions (Figure 3(b)) between the KM and PWK were almost found to have a relatively close topological relationship (Figure 4(a)). Furthermore, five loci with KM/WSB or KM/PWK topology were randomly selected, and the phylogenetic trees were confirmed by Mega software (Figure 4(b)).

## 4. Discussion

Because the KM mouse is used regularly in pharmaceutical and genetic studies, its detailed genetic structure is of great value to the research community. In this study, we sequenced the genome of a male B6-Chr1^KM^ mouse, in which Chr 1 was derived from one KM mouse. The detailed sequence analysis would provide new insights into the application of B6-Chr1^KM^ in biomedical research.

In this study, we identified 479,956 SNPs and 96,679 indels on Chr 1, of which 8.1% did not exist in the MGP and dbSNP142 data sets, indicating that these variants were unique to the B6-Chr1^KM^ mice. Therefore, these variants can be used as unique genetic markers for the genetic quality control of KM mouse. As the most common types of genetic variants, SNPs and indels have been increasingly recognized as having a wide range of effects on gene functions. Among the variants identified on Chr 1, most were located within intergenic or intronic regions. However, we also identified 474 functional variants (missense variant with SIFT < 0.05, stop gain or loss variant, frameshift variant, and splice donor or acceptor variant) which influenced 155 genes. Additionally, several genes have been identified to be associated with human diseases, making them interesting candidates for further functional studies using KM mouse or our newly build B6-Chr1^KM^ strain. For example, Rd3, which is associated with retinal degeneration, was identified as a missense substitution (A->T) with significant deleterious effects (*p* = 0.02). Previous studies have shown that mice with a homozygous mutation in Rd3 exhibit retinal degeneration at three weeks after birth [15]. We also identified a splice acceptor variant in Lamb3 gene, which is associated with blistering of the skin. The mouse models with homozygous Lamb3 628 G->A showed blistering and erosions after birth [16].

Since KM mouse is originated from Swiss mice, it has been speculated to be contaminated with *M. m. castaneus*. In 1991, the morphological characteristics and isozyme polymorphisms of KM and Swiss mice were evaluated, revealing the presence of distinct genetic differences between them [17]. Comparison of KM mouse with wild mice of *M. m. castaneus* captured in Kunming has revealed that the former is more closely related to *M. m. domesticus* than to *M. m. castaneus*. Conversely, contamination of KM mouse by *M. m. castaneus* has been previously demonstrated using the isozyme test [18]. In 2003, the results of a study involving the detection of isozyme polymorphisms also supported the grouping of KM and Swiss mice with *M. m. domesticus* and not with *M. m. musculus* or *M. m. castaneus* [2]. However, it has not yet been confirmed whether KM mouse contains part of the genome of *M. m. musculus* or *M. m. castaneus*. Therefore, high resolution studies of Chr 1 of KM mouse by next-generation sequencing may clarify whether these mice were originated from Swiss mice and/or other mice. Our sequence similarity analysis provided substantial evidence that KM mouse was derived from *M. m. domesticus*, which means that Swiss mice were their ancestor. Both 100 kb blocks and sliding window similarity analysis demonstrated that the Chr 1 of KM mouse was largely composed of *M. m. domesticus* sequences with the rest may derive from *M. m. musculus* or *M. m. castaneus*. Therefore, further analysis is needed to determine the proportion of each subspecies contribution to the Chr 1 of KM mouse.

With the increasing number of whole genome data sets, the reconstruction of phylogenetic trees at a genomic scale has become feasible. Exploration of these large data sets has revealed that there may be discordance among the topologies in different genomic regions [19, 20]. Although these differences may be caused by incorrect estimations of gene genealogies, incongruent gene trees can also be attributed to the differing evolutionary histories of different genomic regions, especially for close species or subspecies. Traditionally, there are two types of phylogenetic analysis methods, the consensus method and the total evidence method. Both methods barely quantify the topological discordance across the entire genome. Recently, BCA, which is an improvement upon the consensus method, has been used to statistically quantify the discordance, as well as to generate phylogenetic trees [21]. A few studies using BCA have demonstrated its great potential for the reconstruction of phylogenic trees of mouse subspecies [11, 13, 22]. These studies indicate that BCA is a suitable method to quantify the proportions of Chr 1 sequence in B6-Chr1^KM^ derived from the different subspecies. Through BCA, we found approximately that 90% and 10% of the sequences of Chr 1 were derived from *M. m. domesticus* and *M. m. musculus*, respectively. Although the sequence similarity analysis revealed that there were some regions which had higher sequence similarity with CAST, we did not observed the same results in the BCA. Therefore, we cannot make the conclusion that some of Chr 1 sequence of B6-Chr1^KM^ came from CAST which represent *M. m. castaneus.* While for PWK, highly conserved genomic regions (Figure 3(b)) with KM aligned well with the BCA results (Figure 4(a)). Thus, from both analyses, we can make the conclusion that Chinese KM mouse has a mosaic genome structure with sequences predominately derived from *M. m. domesticus* and with at least some of the remaining sequences derived from *M. m. musculus*.

In summary, we presented the analysis of a high-quality genome sequence of the B6-Chr1^KM^. These data allow better understanding of the structure and origin of the genetic variations in the B6-Chr1^KM^ mouse strain, which provides insights into the utility of this mouse strain and the KM outbred stock for further biomedical research and the study of complex diseases.

## Figures and Tables

**Table 1:** Predictions of functional consequences of SNPs and indels.

Consequences	SNPs	Novel SNPs	Indels	Novel indels
splice_donor_variant	29	9	2	0
splice_acceptor_variant	11	0	4	0
stop_gained	19	8	1	1
frameshift_variant	0	0	22	7
stop_lost	5	1	1	0
start_lost	11	3	2	0
missense_variant	2378	486	—	0
inframe_insertion	0	0	28	2
inframe_deletion	0	0	26	2
splice_region_variant	1117	63	244	18
synonymous_variant	4238	281	0	0
stop_retained_variant	3	0	0	0
coding_sequence_variant	1	0	1	0
mature_miRNA_variant	4	2	2	0
5_prime_UTR_variant	1210	86	198	31
3_prime_UTR_variant	6563	484	1617	191
non_coding_transcript_exon_variant	11,955	640	2140	290
intron_variant	575,013	36,838	139,815	21,458
NMD_transcript_variant	42,052	2609	10,291	1372
non_coding_transcript_variant	143,110	8770	32,985	5190
upstream_gene_variant	96,888	8357	24,321	3992
downstream_gene_variant	93,184	5752	23,557	3390
intergenic_variant	224,557	13,198	48,568	8312

Consequences were predicted using Ensembl VEP and gene models from Ensembl version 76. Novel SNPs or indels are defined as variants that were not in MGP and dbSNP142 data sets.

**Table 2:** List of human disease-associated genes with loss of function variants in B6-Chr1^KM^ Chr 1.

Gene	Ensembl ID	Variant type	Phenotype	OMIM ID
Col4a3	ENSMUSG00000079465	Frameshift	Alport syndrome, autosomal dominant	*104200*
			Alport syndrome, autosomal recessive	*203780*
			Hematuria, benign familial; BFH	*141200*
Fn1	ENSMUSG00000026193	Frameshift	Glomerulopathy with fibronectin deposits 2; GFND2	601894
			Plasma fibronectin deficiency	614101
Pde6d	ENSMUSG00000026239	Splice donor	Joubert syndrome 22; JBTS22	615665
Hmcn1	ENSMUSG00000066842	Frameshift	Macular degeneration, age-related, 1; ARMD1	603075
Cd244	ENSMUSG00000004709	Stop gain; splice donor	Rheumatoid arthritis; RA	180300
Rab3gap2	ENSMUSG00000039318	Splice acceptor, missense	Martsolf syndrome	212720
			Warburg micro syndrome 2; WARBM2	614225
Lamb3	ENSMUSG00000026639	Splice acceptor	Amelogenesis imperfecta, type IA; AI1A	104530
			Epidermolysis bullosa, junctional, Herlitz type	*226700*
			Epidermolysis bullosa, junctional, non-Herlitz type	*226650*
Dst	ENSMUSG00000026131	Missense	Epidermolysis bullosa simplex, autosomal recessive 2; EBSB2	615425
			Neuropathy, hereditary sensory and autonomic, type VI; HSAN6	*614653*
Ercc5	ENSMUSG00000026048	Missense	Xeroderma pigmentosum, complementation group G; XPG	278780
Casp8	ENSMUSG00000026029	Missense	CASPase 8 deficiency	*607271*
			Dermatitis, atopic	*603165*
Tmem237	ENSMUSG00000038079	Missense	Joubert syndrome 1; JBTS1	213300
			Joubert syndrome 14; JBTS14	614424
Bard1	ENSMUSG00000026196	Missense	Breast cancer	*114480*
Bcs1l	ENSMUSG00000026172	Missense	Bjornstad syndrome; BJS	262000
			Gracile syndrome	*603358*
			Leigh syndrome; LS	256000
			Mitochondrial complex III deficiency, nuclear type 1; MC3DN1	124000
Obsl1	ENSMUSG00000026211	Missense	Three M syndrome 2; 3 M2	612921
Tm4sf20	ENSMUSG00000026149	Missense	Specific language impairment 5; SLI5	615432
Dis3l2	ENSMUSG00000053333	Missense	Perlman syndrome; PRLMNS	267000
Chrng	ENSMUSG00000026253	Missense	Multiple pterygium syndrome, Escobar variant; EVMPS	265000
			Multiple pterygium syndrome, lethal type; LMPS	253290
Ugt1a1	ENSMUSG00000089960	Missense	Crigler-Najjar syndrome, type I	218800
			Crigler-Najjar syndrome, type II	606785
			Gilbert syndrome	143500
			Hyperbilirubinemia, transient familial neonatal; HBLRTFN	237900
Steap3	ENSMUSG00000026389	Missense	Anemia, hypochromic microcytic, with iron overload 2; AHMIO2	615234
Ube2t	ENSMUSG00000026429	Missense	Fanconi anemia, complementation group T; FANCT	616435
Ppox	ENSMUSG00000062729	Missense	Porphyria variegata	*176200*
Ackr1	ENSMUSG00000037872	Missense	Malaria, susceptibility to	611162
Spta1	ENSMUSG00000026532	Missense	Elliptocytosis 2; EL2	130600
			Pyropoikilocytosis, hereditary; HPP	266140
			Spherocytosis, type 3; SPH3	*270970*
Ephx1	ENSMUSG00000038776	Missense	Epoxide hydrolase 1, microsomal; EPHX1	132810
			Hypercholanemia, familial; FHCA	607748
			Preeclampsia/eclampsia 1; PEE1	189800
Rd3	ENSMUSG00000049353	Missense	Leber congenital amaurosis 12; LCA12	*610612*
Cd46	ENSMUSG00000016493	Missense	Hemolytic uremic syndrome, atypical, susceptibility to, 2; AHUS2	612922
Cr2	ENSMUSG00000026616	Missense	Immunodeficiency, common variable, 2; CVID2	240500
			Immunodeficiency, common variable, 7; CVID7	614699
			Systemic lupus erythematosus, susceptibility to, 9; SLEB9	610927

OMIM: online Mendelian inheritance in man. Numbers in italic in OMIM ID column indicate that these diseases have mouse models. Human disease-related phenotypes come from “Human-Mouse: Disease Connection” database (http://www.informatics.jax.org/humanDisease.shtml) in Mouse Genome Informatics website.

## Data Access

All raw reads were submitted to NCBI Sequence Read Archive under the Accession no. SRR2954707 associated with BioProject Accession no. PRJNA298468 and BioSample Accession no. SAMN04159475.
